# Different semantic and affective meaning of the words associated to physical and social pain in cancer patients on early palliative/supportive care and in healthy, pain-free individuals

**DOI:** 10.1371/journal.pone.0248755

**Published:** 2021-03-31

**Authors:** Eleonora Borelli, Sarah Bigi, Leonardo Potenza, Fabrizio Artioli, Sonia Eliardo, Claudia Mucciarini, Katia Cagossi, Giorgia Razzini, Antonella Pasqualini, Fausta Lui, Fabio Ferlazzo, Massimiliano Cruciani, Eduardo Bruera, Fabio Efficace, Mario Luppi, Cristina Cacciari, Carlo Adolfo Porro, Elena Bandieri

**Affiliations:** 1 Department of Biomedical, Metabolic and Neural Sciences, University of Modena and Reggio Emilia, Modena, Italy; 2 Center for Neuroscience and Neurotechnology, University of Modena and Reggio Emilia, Modena, Italy; 3 Department of Linguistic Sciences and Foreign Literatures, Catholic University of the Sacred Heart, Milan, Italy; 4 Hematology Unit and Chair, Azienda Ospedaliera Universitaria di Modena, Modena, Italy; 5 Department of Medical and Surgical Sciences, University of Modena and Reggio Emilia, Modena, Italy; 6 Oncology and Palliative Care Units, Civil Hospital Carpi, USL, Carpi, Italy; 7 Department of Psychology, Sapienza University, Rome, Italy; 8 Palliative Care & Rehabilitation Medicine, UT MD Anderson Cancer Center, Houston, Texas, United States of America; 9 Health Outcomes Research Unit, Italian Group for Adult Hematologic Diseases (GIMEMA), Rome, Italy; University of Technology Sydney, AUSTRALIA

## Abstract

Early palliative/supportive care (ePSC) is a medical intervention focused on patient’s needs, that integrates standard oncological treatment, shortly after a diagnosis of advanced/metastatic cancer. ePSC improves the appropriate management of cancer pain. Understanding the semantic and emotional impact of the words used by patients to describe their pain may further improve its assessment in the ePSC setting. Psycholinguistics assumes that the semantic and affective properties of words affect the ease by which they are processed and comprehended. Therefore, in this cross-sectional survey study we collected normative data about the semantic and affective properties of words associated to physical and social pain, in order to investigate how patients with cancer pain on ePSC process them compared to healthy, pain-free individuals. One hundred ninety patients and 124 matched controls rated the Familiarity, Valence, Arousal, Pain-relatedness, Intensity, and Unpleasantness of 94 words expressing physical and social pain. Descriptive and inferential statistics were performed on ratings in order to unveil patients’ semantic and affective representation of pain and compare it with those from controls. Possible effects of variables associated to the illness experience were also tested. Both groups perceived the words conveying social pain as more negative and pain-related than those expressing physical pain, confirming previous evidence of social pain described as worse than physical pain. Patients rated pain words as less negative, less pain-related, and conveying a lower intense and unpleasant pain than controls, suggesting either an adaptation to the pain experience or the role played by ePSC in improving patients’ ability to cope with it. This exploratory study suggests that a chronic pain experience as the one experienced by cancer patients on ePSC affects the semantic and affective representation of pain words.

## Introduction

Early palliative/supportive care (ePSC) is a medical intervention integrated with standard oncological care, shortly after a patient is diagnosed with advanced/metastatic cancer. ePSC focuses on the patients’ needs and is meant to offer the best possible quality of life to cancer patients and their caregivers. Indeed, in the last years, several randomized controlled trials have shown that ePSC may lead to a better quality of life and decreased depression [1–6], lower rates of chemotherapy near death [1,3,7], increased prognosis awareness [5,8], and longer survival [1,2,9].

According to the World Health Organization, the beneficial effects of ePSC arise from an “early identification and impeccable assessment and treatment of pain and other problems, physical, psychosocial and spiritual” [10]. Pain is among the most common and distressing symptoms described by cancer patients. Pain severity independently predicts survival beyond established biomedical parameters [11]. Cicely Saunders coined the expression “total pain” to capture the complexity of cancer pain that results from a combination of physical and psychosocial sufferings simultaneously caused by the disease, oncologic treatments, and patients’ thoughts and feelings [12]. Although physical pain can be pharmacologically controlled, pain relief still represents a challenge in oncology [13] also because of a general underestimation of the multidimensional origin of cancer pain and a tendency to focus only on its physical component [14]. The multidisciplinary approach standing behind ePSC has proved to be effective in the assessment and management of total pain in cancer patients [13].

Establishing how much pain, and the type of pain, a cancer patient has experienced and/or is currently experiencing derives from the verbal descriptions produced by patients during doctor-patient verbal exchanges (e.g., [15,16]). This is the rationale whereby ePSC has identified verbal communication skills as fundamental to evaluate patients’ total pain [17,18]. To aid the increase of ePSC communication skills in oncology, guidelines for an optimal oncological communication (e.g., SPIKES, NURSE, www.vitaltalk.org) have been proposed [19].

It is well established that suboptimal communication can negatively affect clinical outcomes [20,21]. The oncological literature has shown that cancer patients still have unmet communication needs, especially when physicians do not attend patients’ emotional needs [22]. Thus, understanding what patients really mean when they verbally describe their pain as well as the affective impact of these words is a key step for a better assessment of what patients are experiencing and for a general improvement of doctor-patient communication [23].

### Physical and social pain

Undeniably physical pain represents only one of the two sides of the pain experience of oncological patients. The psychological and social suffering associated with this illness represents the other side [12].

Macdonald & Leary [24] coined the expression “social pain” to convey the feelings of pain emerging in response to threats to and/or losses of social connection, e.g., social rejection, social exclusion, or loss of significant others. This definition partly overlaps with a general notion of psychological pain [25,26]. Indeed, most authors consider social pain one of the main subtypes of psychological pain, defining it as the psychological pain experienced in case of social incentive loss [27–30]. Eventually, social pain has also been described as a stressor that can exacerbate psychological pain [31]. Across languages, situations associated with social distress are often identified with physical pain terms: for instance, “This is a painful situation”, “This event/person is a headache”, “She’s a pain in the neck” [24,32,33]. This use of physical pain words to convey social pain may not simply reflect a metaphorical transfer. Indeed, several studies suggest that social pain and physical pain may interact at different levels. According to Zhang et al. [34], the perception of physical pain is increased by a condition of social pain [35–37] and decreased by either a reduction of social pain [38] or by social support [37,39,40]. Similarly, physical pain can exacerbate feelings of social pain [41,42], even without actual experiences of exclusion [43]. Moreover, individuals who are more sensitive to physical pain are also more sensitive to social pain [44].

Social pain is commonly experienced and reported by cancer patients because of the impact of the diagnosis on their social life. This suggests that this component of the pain experience should not be ignored and that the assessment and management of patients’ physical and social pain should not be separated.

### Words of pain

Albeit words represent the main tool we use to describe physical and psychosocial pain, translating these experiences into words is difficult and often frustrating given the subjective complexity of pain. To overcome this problem in the clinical practice, several medical doctors proposed to administer pain questionnaires to assess the pain experienced by patients. For instance, the McGill Pain Questionnaire [45] is based on a finite set of descriptors that should capture and categorize the facets of the pain experience. However, the predictive value of these descriptors remains to be fully demonstrated (for an overview, see ref. [46]).

#### The emotional and psycholinguistic characteristics of words

An extensive psychological literature has shown that the emotional content of words, as well as many other word characteristics (e.g., their familiarity), affects the ease with which we comprehend words and sentences [47,48]. However, only a few studies specifically explored these characteristics in pain words and none involved patients indeed experiencing a pain condition. In a previous study from our group conducted on healthy young participants [49], we investigated the relationships among emotional content, psycholinguistic characteristics, and some variables specifically associated to pain in 512 Italian words conveying physical and social pain.

The main aim of this cross-sectional survey study was to unveil the semantic and affective representation of physical and social pain in a sample of patients with cancer pain on ePSC. To investigate wheteher and the extent to which their condition of chronic pain modulates this representation, we compared the data emerging from patients with those of healthy, pain-free, matched individuals. To this aim, we asked patients and controls to rate the semantic and affective characteristics of 94 Italian words conveying physical pain (e.g., *lancinante*, piercing) or social pain (e.g., *rassegnazione*, resignation).

Symptom burden, unmet needs, level of spirituality and of hope are known to affect the ability of cancer patients to cope with pain [50–56]. Therefore, the second aim of this study was to explore whether these variables correlate with pain word ratings.

## Methods

### Participants

One-hundred ninety advanced cancer patients in ePSC (106 women; age range: 33–88, mean: 67, SD: 11.7) were consecutively recruited from March 2018 to March 2019 at the Oncology and Palliative Care Unit, Carpi Civil Hospital, within the Local Health Unit in Modena. Since 2012, an interdisciplinary working group operates at the Oncology and Palliative Care Unit of the Carpi Civil Hospital. This group integrates primary oncologist specialists with an ePSC team in order to provide comprehensive symptom management and psychosocial, spiritual, and emotional support to cancer patients and their relatives, from the time of diagnosis of advanced/metastatic disease, onward. Specific guidelines [1,13,57] ensure a uniform and reproducible intervention. Although the specific care provided by the ePSC team is centered on the patient and family’s needs, these guidelines include the evaluation and management of the common symptoms associated with advanced/metastatic disease, namely pain, gastrointestinal symptoms (anorexia and weight loss, nausea and vomiting, and constipation and diarrhea), fatigue, sleep, and mood disturbances (anxiety and depression). The ePSC team also provides assistance with treatment choice, assistance of patients, and family caregivers in coping with a life-threatening illness. Patients with advanced cancer are defined as those with distant metastases (i.e., in case of solid tumors), late-stage disease and/or with a prognosis of 6–24 months. The in-patient ePSC team follows each patient on a regular basis, at least once a week.

The eligibility criteria for patients to be included in the study were to have an advanced/metastatic cancer diagnosis with severe cancer pain, willingness to participate, Italian as mother tongue (or at least 15 years spent in Italy), age ≥ 18 years old, no cognitive impairment (Mini Mental State Examination > 24), no history of psychiatric or neurological disorders, and no current use of psychoactive medications.

The control group consisted of 124 healthy, pain-free participants (72 women; age range: 41–91, mean: 63.7, SD: 11.3) recruited in several community and senior centers in the Modena district from June 2018 to September 2019. The recruitment occurred through flyers distributed in local community and senior centers (e.g., blood donor association, lawn bowling club, pensioners’ recreation places). The flyers reported the starting of a study on pain language in clinical and non-clinical settings based on questionnaires and indicated the presence of a researcher recruiting healthy volunteers to participate in the study. The eligibility criteria for controls to be included in the study were willingness to participate, absence of past or actual diagnosis of cancer and/or other chronic pain diseases and numerical rating scale equal to zero during the previous 24 hours, at the time of enrolment, no first-degree relatives with cancer, Italian as mother tongue (or at least 15 years spent in Italy), age ≥ 18 years old, no cognitive impairment (Mini Mental State Examination > 24), no history of psychiatric or neurological disorders, and no current use of psychoactive medications.

The two overall groups were matched for gender, age, and educational level.

Participants provided signed informed consent prior to data collection. The study was performed in accordance with the ethical standards of the 2013 Declaration of Helsinki and was approved by the Ethics Committee of Modena (N. 255/17).

### Materials

A consensus group of five clinicians (3 oncologists, 2 hematologists) with a long-term expertise in ePSC was asked to select the pain words most frequently used in doctor-patient communication based on the dataset of 512 physical and social pain words collected in our previous study (Word of Pain, WOP; [49]). In the WOP dataset, the physical and social pain words were selected through an extraction procedure typical of the computational linguistic research. This procedure is based on the assumption that the lexicon is a metrical space in which words are separated by distances that depend on the degree of semantic similarity between words measured through their statistical co-occurrence distribution in texts. We used the word *dolore* (pain) as an anchor point and selected the content words co-occurring with it in a window of 25 words to the left and 25 words to the right of the word *dolore* in linguistic corpora, medical dictionaries, blogs, and pain questionnaires.

The consensus group received an online survey with a brief description of the aim of the study and was asked to rate how frequent was the use of each word of the dataset in doctor-patient communication in oncology. The rating scale reported below each word went from Not frequent at all (1) to Extremely frequent (7). For each word, the mean, median, standard deviation, and internal consistency (Cronbach’s alpha) of the ratings of the consensus group were calculated. Before starting the online survey, we established a maximum of 100 words to be included in the study to limit the cognitive load of the task. We selected the words with the highest median value and rating consistency. This led to a list of 94 words (adjectives, nouns, verbs), of which 75 associated to physical pain and 19 to social pain (see S1 Table for the stimulus list). Since some pain words in our original normed database [49] could be used to refer to both physical and social pain, we asked 67 different participants (43 women; age range: 19–40, mean age: 33 years, SD = 5.1) to decide whether these words predominantly referred to physical or social pain (see WOP for the percentages).

The 94 words used in this study were pseudo-randomly distributed over four questionnaires to be administered to participants. To reduce unpredictable effects of random word orders, the same word list was repeated for each of the ten variables of interest. Each questionnaire started with a section requiring demographic information (gender, year of birth, education) followed by an introduction explaining the aim of the study and the duration (approximately 20 minutes per questionnaire). Each word list was preceded by a definition of the variable to be rated and an explanation on how to use the Likert (or VAS) scale. In order to reduce the support of the interviewer and possible biasing effects, two examples of words rated with extreme values were provided (see the original Italian instructions and their English translation in S1 Text).

Patients and controls were asked to rate the semantic and affective characteristics of each word based on variables currently used in psycholinguistic and affective studies about the semantic representation of words [58] and in pain studies [49]. Specifically, for each word we asked: (i) Familiarity (*Familiarità*; i.e., the frequency with which a word occurs in everyday life) [59]; (ii) Valence (*Valenza*; i.e., the extent to which the word conveys a positive or negative affect) [60]; (iii) Arousal (*Attivazione*; i.e., the extent to which the word content conveys a physiologically alerting or calming state) [60,61]; (iv) Pain-relatedness (*Grado di associazione al dolore*; i.e., how much the word is related to pain) [49]; (v) Intensity (*Intensità*; i.e., how strong is the pain depicted by the word); and (vi) Unpleasantness (*Spiacevolezza*; i.e., how disturbing is the pain depicted by the word). Intensity and Unpleasantness respectively refer to the sensory-discriminative and affective-motivational dimensions of pain [62]. We decided not to collect ratings about pain impact because studies on pain and language mainly focus on the intensity and unpleasantness of pain and also to avoid overloading participants’ fatigue.

Most of the variable was rated using a Likert scale. Specifically, the scales went from Not at all familiar (1) to Extremely familiar (7); Extremely negative (-3) to Extremely positive (+3); Not at all arousing (1) to Extremely arousing (7); Not at all associated to pain (1) to Extremely associated to pain (7); Not at all unpleasant (1) to Extremely unpleasant (10); and Not at all intense (1) to Extremely intense (10). Participants were instructed to select the option "I don’t know" when they did not know the word meaning.

### Assessment of individual differences

In order to evaluate the impact of symptom burden, unmet needs, spirituality, and hope on the meaning and affective correlates of pain related language, we administered the following scales commonly used in the clinical ePSC practice. The Edmonton Symptom Assessment Scale (ESAS) evaluates the symptom burden in patients with advanced cancer admitted to a palliative care unit [50]. The Italian version [51] consists of nine numerical rating scales that assess pain, fatigue, nausea, depression, anxiety, drowsiness, appetite, sense of well-being, and shortness of breath, with higher scores denoting higher symptom burden.

The Needs Evaluation Questionnaire (NEQ) assesses hospitalized cancer patients’ needs [52]. It is composed by 23 items that assess the needs of information concerning diagnosis, prognosis, examinations and treatments, communicative and relational needs, assistance, treatment, hospital structure, financial and support needs. The higher the number of positive responses, the higher the number of unmet needs.

The System of Belief Inventory (SBI-15R) is a self-administered scale assessing patient’s religiousness and spirituality [53]. The Italian version [54] is composed by two subscales referring to religious beliefs and practices (Beliefs subscale) and to the social support provided by the religious community (Support subscale). Each item is rated on a 4-point Likert scale ranging from “Strongly disagree” to “Strongly agree” or from “Never” to “Always”, with higher scores denoting higher levels of adherence to a religious beliefs system.

The Hope Herth Index (HHI) provides a measure of hope in cancer patients investigating the inner sense of temporality and future, inner positive readiness and expectancy, and interconnectedness with self and others [55]. The Italian version of HHI [56] is composed by 12 items rated on a 4-point Likert scale ranging from “Strongly disagree” to “Strongly agree”, with higher scores denoting greater hope.

### Procedure

The patients completed the word rating questionnaires at a time of their preference and availability during the weekly appointments at the Oncology and Palliative Care Unit in Carpi. They were free to complete all the word questionnaires at once or in separate sessions. A previously trained nurse gave the patient one questionnaire at a time and read with her/him the introduction and instructions about the variables to be rated. Then, the questionnaire was completed in a self-paced way by the patient. Patients also self-completed the ESAS, NEQ, SBI-15R, and HHI scales at their pace.

Controls completed the word rating questionnaires where they were recruited. They were free to complete all the word questionnaires at once or in separate sessions. A researcher gave the volunteer one questionnaire at a time and read with her/him the introduction and instructions about the variables to be rated. Then, the questionnaire was completed in a self-paced way by the control.

All data were collected from March 2018 to November 2019.

### Study design and data analysis

A cross-sectional survey study was preferred to a qualitative approach in order to enable sampling and analysis of a large dataset and to investigate relation between these data and other quantitative measures in future studies.

In line with other psycholinguistic studies, to exclude inaccurate data due to fatigue of lack of attention, we set a cutoff of no more than 30% of responses “I don’t know” and/or no more than 30% of missing responses to include a participant’s questionnaire in the study. Because the scoring procedure of the ESAS, NEQ, SBI-15R, and HHI scales is based on summing up each item’s score, we also excluded from the analyses the scales with at least a missing item. This procedure led to the exclusion of 17 patients for whom none of the questionnaires met the cutoff. No control participant was eliminated using the same procedure. The two final samples consisted of 173 patients (98 women; range age: 33–88, mean: 66, SD: 11.5) and 124 controls (72 women; range age: 41–91, mean: 63.7, SD: 11.3).

To test between-group differences (where the group variable is defined by the status of “patient” or “control”) in the ratings of each variable and the differences between physical and social pain words within and between groups, the mean rating for each word for each variable was standardized on a 7-point scale. Valence scores were reversed in order to associate lower scores to lower negativity and higher scores to higher negativity.

A three-way mixed ANOVA was performed with the factors Group (Patients vs. Controls), Variable (Familiarity vs. Valence vs. Arousal vs. Pain-relatedness vs. Intensity vs. Unpleasantness), and Type of pain (Physical pain words vs. Social pain words). Group was the only between-subject factor. We comment only significant effects related to Type of pain and Group.

Six Bonferroni-corrected multiple regression analyses were carried out to investigate whether individual differences in patients’ age, education, symptom burden, unmet needs, spirituality, and hope, were associated to the patients’ ratings.

The analyses were performed using Statistica software (TIBCO Software Inc., 2018). All data relevant to the study are included in the article or uploaded as supplementary information or available upon request.

## Results

The demographic characteristics of patients and controls are reported in Table 1.

**Table 1:** Demographic characteristics of participants.

	Patients (n = 173)		Controls (n = 124)		*p*
	Mean	SD	Mean	SD	
Gender	M = 75; W = 98		M = 52; W = 72		.81
Age (yrs)	66	11.5	63.7	11.3	.08
Education (yrs)	11	3.7	11.2	3.6	.58

Patients and controls’ demographic characteristics once excluded patients’ questionnaires that did not match the inclusion criteria (see Data Analysis section). N, sample size; SD, Standard deviation; *p*, *p*-value; M, Men; W, Women; Yrs, Years.

Table 2 shows descriptive statistics for Familiarity, Valence, Arousal, Pain relatedness, Intensity, and Unpleasantness. On average, patients and controls knew 95% and 97% of the words, respectively (see S1 Table for the percentages of Unknown response of each stimulus). Predictably, the number of Unknown responses negatively correlated with the years of education for both groups, once the effect of age was controlled for (patients: r = -.48, p < .001; controls: r = -.22, p = .017).

**Table 2:** Descriptive statistics of participants’ ratings in each variable for physical and social pain words.

		Fam			Val			Aro			Pain-rel			Int			Unpl		
		M	SD	n	M	SD	n	M	SD	n	M	SD	n	M	SD	n	M	SD	n
**Physical pain words**	**P**	5.2	1.6	11268	4.3	0.5	11267	3.4	0.8	11244	3.6	0.8	11231	4.2	1.3	11245	4.3	1.3	11223
	**C**	5	1.4	8613	5.1	0.8	8607	3.7	1.4	8523	4.9	1	8585	6.7	1.6	8621	6.9	1.7	8603
**Social pain words**	**P**	6	1.1	2904	5.4	0.8	2907	4.9	1	2907	4.9	0.8	2896	6.2	1.3	2905	6.7	1.3	2894
	**C**	5	1.5	2191	6.2	0.9	2190	3	1.9	2168	5.2	1.2	2181	6.8	1.9	2190	7.3	1.9	2184

Fam, Familiarity; Val, Valence, Aro, Arousal; Pain-rel, Pain relatedness; Int, Intensity; Unpl, Unpleasantness; M, Mean; SD, Standard deviation; n, Number of observations; P, Patients; C, Controls.

**Fig 1:**
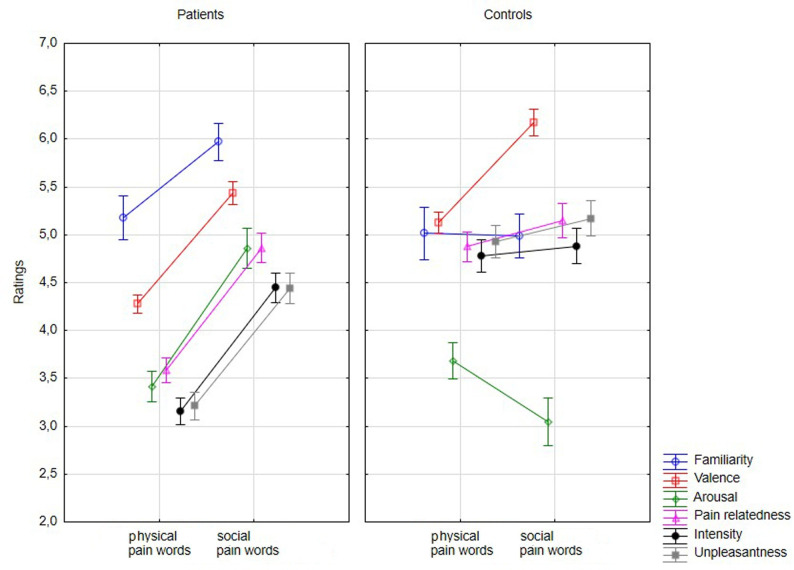
Graphic representation of the three-way mixed ANOVA results.

**Fig 2:**
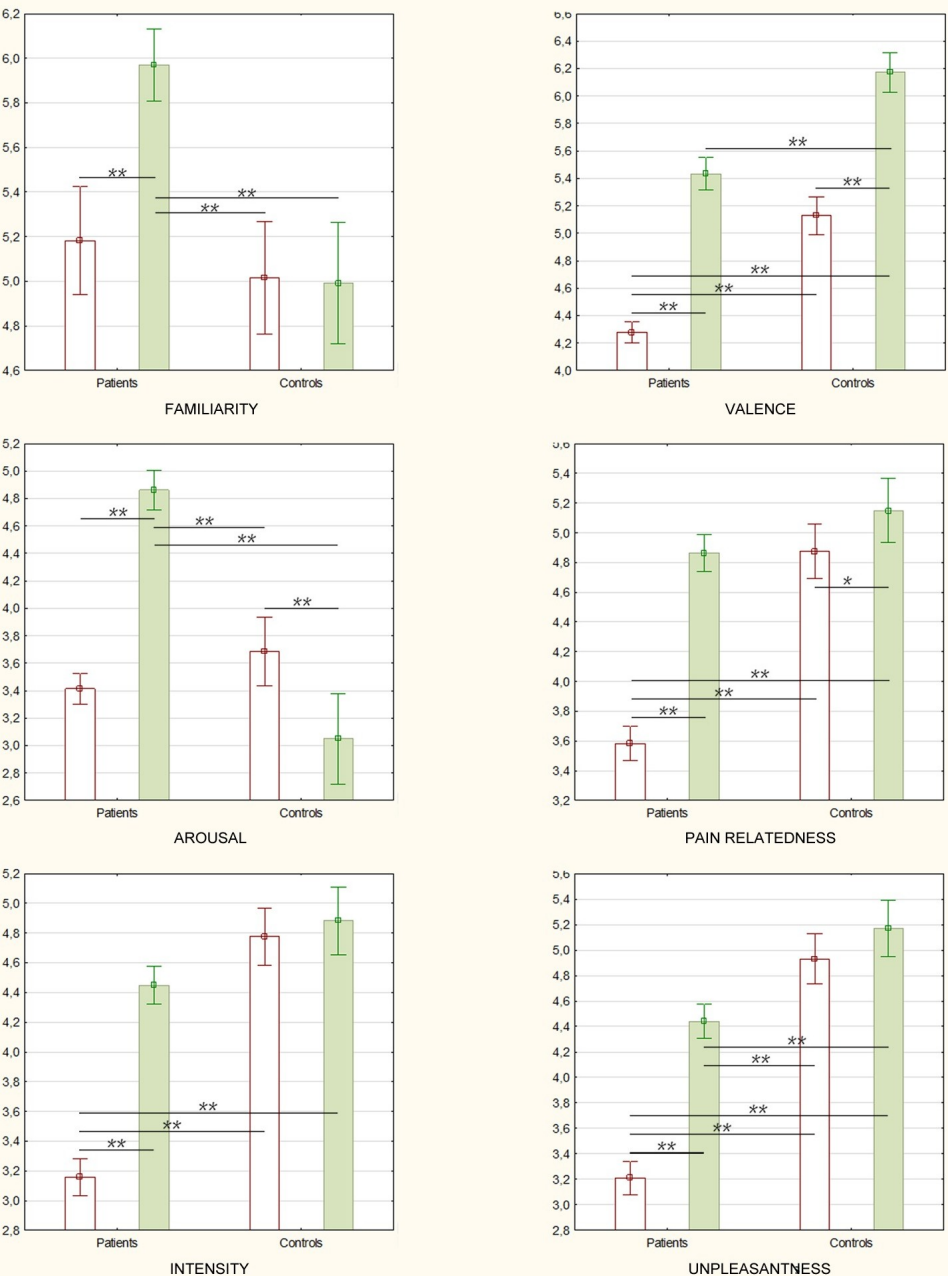
Variables’ mean ratings as a function of group and type of pain. White bars represent physical pain words. Green bars represent social pain words. Error bars represent 95% confidence intervals. * p < .01, ** p < .001.

The effects of Group and Type of pain on each variable were analyzed using a three-way mixed ANOVA (Table 3 and Fig 1). Since the assumption of sphericity was violated, a Greenhouse-Geisser correction was applied. Significant main effects of Group and Type of pain were obtained. Controls rated pain words as more negative, more associated to pain, and conveying a more intense and unpleasant pain than patients. Social pain words obtained higher ratings than physical pain words in all the variables. A significant Type of pain x Group interaction was obtained. Post-hoc Bonferroni tests (Fig 2) showed that patients rated social pain words as significantly more familiar and arousing, but less negative and unpleasant, than controls. Patients also rated physical pain words as significantly less negative, pain related, intense, and unpleasant than controls. Patients rated social pain words as more familiar, negative, arousing, pain related, intense, and unpleasant than physical pain words. Controls rated social pain words as more negative and pain related, but less arousing, than physical pain words.

**Table 3:** Summary of the three-way mixed ANOVA.

	df	F	*p*-value	Partial η^2^
Ratings				
Group	1, 295	30.14	< .001	0.09
Type of pain	1, 295	351.1	< .001	0.54
Variable	2.85, 840.3	151.79	< .001	0.34
Group x Type of pain	1, 295	200.13	< .001	0.4
Group x Variable	2.85, 840.3	81.1	< .001	0.22
Type of pain x Variable	4.12, 1215.6	37.72	< .001	0.11
Group x Type of pain x Variable	4.12, 1215.6	54.18	< .001	0.16

Table 4 shows the descriptive statistics of ESAS (including the single item on pain reported separately), NEQ, SBI-15R, and HHI scores for patients. The multiple linear regression analyses with symptom burden, needs, spirituality, hope, and demographical variables (age and education) as predictors, revealed that the model significantly predicted patients’ pain words ratings for Familiarity [F(7, 126) = 28.459, p < .001, R^2^ = .61], with Age and Education as significant predictors (B = - .03, p = .006; B = .25, p < .001, respectively); Valence [F (7, 126) = 5.922, p < .001, R^2^ = .25], with ESAS, SBI-15R Belief, and SBI-15R Support measures as significant predictors (B = .02, p < .001; B = -.07, p < .001; B = .03, p = .002, respectively); and Unpleasantness [F(7, 126) = 3.703, p = .001, R^2^ = .17], with ESAS and ISH as significant predictors (B = .02, p = .005; B = .06, p = .004, respectively).

**Table 4:** Descriptive statistics of ESAS, NEQ, SBI-15R, and HHI.

	Patients	
	Mean	SD
ESAS	17.7	12.7
ESAS–pain item	2	2.3
NEQ	9.6	3.1
SBI-15R Belief subscale	7.1	5
SBI-15R Support subscale	19.3	8.7
SBI-15R Total score	26.5	13.1
HHI	36.9	4.7

Patients’ scores once excluded patients’ scales that did not match the inclusion criteria (see Data Analysis paragraph). SD, standard deviation; *p*, *p*-value. NEQ refers to the number of positive responses.

## Discussion

This exploratory cross-sectional survey study revealed important differences in the semantic and affective representations of pain words in cancer patients on ePSC and healthy, pain-free, matched individuals. The study also highlighted important differences between physical and social pain words. Specifically, both groups perceived social pain words as significantly more negative and more associated to pain, replicating what was found in our previous study on healthy young participants [49]. This result is in line with studies documenting that social pain is considered as much, if not more, threatening and important as physical pain [63,64], a connotation that has also been described for psychological pain.

Unexpectedly, despite the fact that differently from healthy, pain-free controls, oncological patients experience severe pain, they rated both physical and social pain words as less negative than controls. One possibility is that this may reflect a long standing and suffered adaptation to illness and to the correlated pain experience. Another possibility, which should be further explored by specific studies, is that the ePSC treatment received by patients improved their ability to cope with the affective impact of illness leading to a perceived decreased negativity of pain words.

Patients rated social pain words as more negative, more arousing, more pain related, and conveying a more intense and unpleasant pain than physical pain words. This may reflect the fact that ePSC is first devised to treat physical pain at a shorter lag via analgesic therapy [65]. In contrast, treating psychosocial pain requires a long-standing ePSC intervention [66–69]. Notably, Yoong et al. [70] found that in the ePSC patients, the attempt to cope with social aspects of cancer is prominent throughout all the visits and the illness trajectory, compared to other needs that are mostly focused and solved in the initial visits.

Oncological patients considered the words associated to social pain as more arousing than those expressing physical pain. In contrast, healthy, pain-free participants considered physical pain words as more arousing than social pain words. This group difference may reflect the threatening connotation of cancer that inspired the ratings of physical pain words in controls. This threatening connotation is less relevant to oncological patients in ePSC, presumably because physical pain is pharmacologically controlled.

Differently from controls, patients rated social pain words as more familiar than physical pain words. Admittedly, we do not have an explanation for this result. One possibility is that the higher familiarity of these words may reflect their higher relevance to patients. Another, not alternative, possibility is that it may reflect the relevance attributed by ePSC to the social aspects of cancer leading to a more extended use of these words during doctor-patient verbal exchanges.

Notwithstanding the fact that physicians should avoid using medical jargon, large part of the literature on doctor-patient communication reports that, typically, doctors use a lexicon unfamiliar to patients, leading to a discrepancy between the words commonly used by doctors and the meaning actually understood by patients [71,72]. This did not seem to be the case in our study since only 5% of words selected by the consensus group, with long-term expertise in ePSC, were unknown to the patients. Moreover, overall the words received high familiarity ratings (5.7 on average).

The lower the symptom burden, the less negative and unpleasant were pain words for the patients. In addition, the larger the availability of spiritual and religious resources, the less negative were perceived pain words. At the same time, the more the patients needed a community support, the more pain words were negative. This may reflect an important link between community support and illness coping. However, the vast literature about the effects of spiritual beliefs and community support on illness perception is far from presenting a homogenous picture [73]. Some studies found that religious support did not have direct or mediating effects on quality of life [74]. Social interaction is not always reported to be pleasant and supportive, and negative interactions with members of social networks may offset or outweigh beneficial effects [74].

Admittedly, this exploratory cross-sectional survey study has some important limitations. The first is associated to a possible sampling bias. Since healthy controls volunteered to participate in a study generically described as concerning the language used to convey pain experiences, only those having less fear of pain may have participated as it has been observed in experimental pain research [75]. However, in controls recruitment we clarified that the study did not include any painful stimulation. Another possible limitation is that the scales we administered to patients, although including the variables generally monitored in clinical practice [1,13,57], may have not exhaustively assessed all the dimensions of the cancer patients’ suffering. Future studies should analyze a wider range of other variables and include, specifically, a more detailed assessment of social well-being and anxiety/depression, in both the patient and the control groups, to allow a more comprehensive investigation of the between group differences. Finally, the lack of a further control group represented by cancer patients receiving standard oncologic treatment does not allow us to establish whether and how much the between group differences in the semantic and affective representations of pain words are associated to ePSC treatment or reflects the illness experience *per se*. Future studies should be carried out to disentangle the effects of illness and cancer care treatment on the semantics of pain.

In conclusion, to the best of our knowledge, this is the first study showing that a severe illness condition like advanced/metastatic cancer affects pain word representations. Indeed, the semantic and affective representation of the words expressing physical and social pain of oncological patients on ePSC differs from that of matched healthy, pain-free controls. This finding is supported by the studies showing how pain leads to alterations of physiological and psychological processes of pain perceptions and pain-related behaviors [76,77]. Moreover, a few previous linguistic studies on the literal and figurative language used to convey the cancer experience by patients suggest that the illness experience indeed affects the semantic representation of physical and social pain words [22,78].

Investigating how oncological patients on ePSC perceive the lexicon of pain can be of help in improving the quality of doctor-patient communication and provide doctors with new tools for assessing how much word meaning and affect are shared with their patients. Since language mediates the ways in which individuals experience illness and has the potential to affect its course, suboptimal communication can negatively impact the patients’ clinical outcome [21]. As Semino [22] stated, a scientific approach to words meanings and their affective correlates makes it possible to understand the potential implications of specific linguistic choices in the oncological setting.

## Supporting information

S1 Table List of the original stimuli with their English translation and the percentages of unknown responses for patients and controls. (DOCX)

S1 Text Italian introduction and instructions of the questionnaire and their English translations. (DOCX)
