# Supplementary material for: Different semantic and affective meaning of the words associated to physical and social pain in cancer patients on early palliative/supportive care and in healthy, pain-free individuals
Source: PLoS One. 2021 Mar 31;16(3):e0248755. doi: 10.1371/journal.pone.0248755 (PMC8011738; doi:10.1371/journal.pone.0248755)
Supplement: S1 Table — (DOCX) [file pone.0248755.s001.docx]

***S1 Table.*** *List of the original stimuli with their English translation and the percentages of Unknown responses for patients and controls.*

|  |  |  | Unknown responses % | |
| --- | --- | --- | --- | --- |
| Italian word | English translation | Type of pain conveyed | Patients | Controls |
| Abituale | Habitual | Physical pain | 13.94 | 2.50 |
| Accecante | Dazzling | Physical pain | 13.64 | 2.46 |
| Acuto | Acute | Physical pain | 1.30 | 2.46 |
| Affaticamento | Fatigue | Physical pain | 14.81 | 0.00 |
| Blando | Bland | Physical pain | 16.08 | 2.61 |
| Bruciante | Burning | Physical pain | 23.64 | 15.83 |
| Cancro | Cancer | Physical pain | 0.65 | 0.82 |
| Colica | Colic | Physical pain | 4.32 | 1.69 |
| Compressione | Compression | Physical pain | 18.18 | 4.35 |
| Continuo | Continuous | Physical pain | 11.52 | 2.50 |
| Costante | Constant | Physical pain | 1.21 | 4.17 |
| Costrittivo | Binding | Physical pain | 0.00 | 0.82 |
| Crampiforme | Crampy | Physical pain | 8.02 | 8.47 |
| Crampo | Cramp | Physical pain | 0.61 | 1.67 |
| Cronico | Chronic | Physical pain | 0.65 | 2.46 |
| Debilitante | Debilitating | Physical pain | 16.08 | 9.57 |
| Debole | Weak | Physical pain | 16.36 | 29.17 |
| Diffuso | Widespread | Physical pain | 0.00 | 0.82 |
| Discreto | Discreet | Physical pain | 1.23 | 0.85 |
| Disturbante | Disturbing | Physical pain | 4.94 | 3.39 |
| Dolore | Pain/ache | Physical pain | 9.09 | 0.00 |
| Eccessivo | Excessive | Physical pain | 0.61 | 1.67 |
| Elevato | High | Physical pain | 1.30 | 3.28 |
| Esagerato | Exaggerated | Physical pain | 4.55 | 1.64 |
| Esasperante | Nerve-racking | Physical pain | 1.23 | 0.00 |
| Fastidioso | Annoying | Physical pain | 7.69 | 0.00 |
| Ferita | Wound | Physical pain | 0.62 | 3.39 |
| Feroce | Fierce | Physical pain | 0.61 | 3.33 |
| Fisico | Physical | Physical pain | 0.70 | 0.00 |
| Fisso | Fixed | Physical pain | 0.00 | 0.00 |
| Fitta | Sharp pain | Physical pain | 1.82 | 5.83 |
| Forte | Strong | Physical pain | 0.65 | 0.00 |
| Frequente | Frequent | Physical pain | 3.25 | 4.10 |
| Grande | Great | Physical pain | 0.62 | 0.85 |
| Improvviso | Sudden | Physical pain | 0.00 | 0.85 |
| Inaccettabile | Unacceptable | Physical pain | 0.00 | 0.00 |
| Incessante | Unceasing | Physical pain | 0.61 | 1.67 |
| Incontenibile | Uncontainable | Physical pain | 0.61 | 5.00 |
| Indefinibile | Indefinable | Physical pain | 0.00 | 0.00 |
| Indicibile | Unspeakable | Physical pain | 0.00 | 0.82 |
| Indolenzimento | Soreness | Physical pain | 0.00 | 0.85 |
| Infiammazione | Inflammation | Physical pain | 0.00 | 0.85 |
| Inimmaginabile | Unimaginable | Physical pain | 0.00 | 0.00 |
| Insistente | Insistent | Physical pain | 2.10 | 0.87 |
| Insopportabile | Unbearable | Physical pain | 7.88 | 6.67 |
| Insostenibile | Unsustainable | Physical pain | 10.91 | 4.17 |
| Intenso | Intense | Physical pain | 6.49 | 6.56 |
| Intervento | Intervention | Physical pain | 9.88 | 18.64 |
| Intollerabile | Intolerable | Physical pain | 5.59 | 0.87 |
| Irradiato | Radiated | Physical pain | 0.00 | 0.83 |
| Lancinante | Piercing | Physical pain | 7.14 | 1.64 |
| Lieve | Slight | Physical pain | 8.64 | 2.54 |
| Localizzato | Localized | Physical pain | 4.55 | 0.82 |
| Male | Bad | Physical pain | 0.70 | 0.87 |
| Malessere | Malaise | Physical pain | 0.00 | 0.87 |
| Moderato | Moderate | Physical pain | 0.00 | 0.00 |
| Persistente | Persistent | Physical pain | 4.55 | 1.64 |
| Profondo | Deep | Physical pain | 10.49 | 16.95 |
| Prolungato | Extended | Physical pain | 0.00 | 0.87 |
| Pulsante | Button | Physical pain | 15.15 | 7.50 |
| Recidivo | Recidivist | Physical pain | 8.02 | 0.85 |
| Scossa | Shock | Physical pain | 0.00 | 2.61 |
| Severo | Severe | Physical pain | 0.65 | 0.00 |
| Sforzo | Effort | Physical pain | 0.62 | 0.85 |
| Sofferenza | Suffering | Physical pain | 0.00 | 0.87 |
| Soffrire | Suffer | Physical pain | 1.21 | 1.67 |
| Sopportabile | Bearable | Physical pain | 1.21 | 2.50 |
| Stordimento | Daze | Physical pain | 0.65 | 0.82 |
| Tagliare | To cut | Physical pain | 0.00 | 2.54 |
| Tensione | Voltage | Physical pain | 0.00 | 2.61 |
| Tortura | Torture | Physical pain | 0.00 | 4.24 |
| Trauma | Trauma | Physical pain | 1.21 | 4.17 |
| Tremendo | Terrible | Physical pain | 3.25 | 0.82 |
| Tremore | Tremor | Physical pain | 3.09 | 0.85 |
| Violento | Violent | Physical pain | 0.70 | 4.35 |
| Affliggere | To afflict | Psychosocial pain | 0.61 | 0.83 |
| Afflizione | Affliction | Psychosocial pain | 7.14 | 4.92 |
| Angoscia | Distress | Psychosocial pain | 0.00 | 2.54 |
| Angosciante | Distressing | Psychosocial pain | 2.60 | 2.46 |
| Depressione | Depression | Psychosocial pain | 0.00 | 0.87 |
| Deprimente | Depressing | Psychosocial pain | 0.00 | 0.85 |
| Disagio | Discomfort | Psychosocial pain | 0.00 | 0.00 |
| Distacco | Detachment | Psychosocial pain | 0.00 | 0.00 |
| Lacrima | Tear | Psychosocial pain | 1.95 | 2.46 |
| Lutto | Mourning | Psychosocial pain | 0.70 | 1.74 |
| Morire | To die | Psychosocial pain | 0.00 | 2.54 |
| Morte | Death | Psychosocial pain | 0.00 | 2.61 |
| Patire | To suffer | Psychosocial pain | 0.00 | 0.83 |
| Paura | Fear | Psychosocial pain | 0.00 | 5.00 |
| Penoso | Painful | Psychosocial pain | 0.00 | 0.82 |
| Rassegnazione | Resignation | Psychosocial pain | 0.00 | 1.69 |
| Strazio | Torment | Psychosocial pain | 0.00 | 0.00 |
| Stress | Stress | Psychosocial pain | 0.61 | 0.83 |
| Tristezza | Sadness | Psychosocial pain | 0.00 | 0.00 |
