# Supplementary material for: Different semantic and affective meaning of the words associated to physical and social pain in cancer patients on early palliative/supportive care and in healthy, pain-free individuals
Source: PLoS One. 2021 Mar 31;16(3):e0248755. doi: 10.1371/journal.pone.0248755 (PMC8011738; doi:10.1371/journal.pone.0248755)
Supplement: S1 Text — (DOCX) [file pone.0248755.s002.docx]

***S1 Text.*** *Italian introduction and instructions of the questionnaire and their English translations.*

ID/*ID*:

Data/*Date*:

Anno di nascita/*Year of birth*:

Sesso/*Gender*:

Scolarità/*Education*:

Buongiorno.

Il presente questionario fa parte di uno studio sul dolore e si propone di raccogliere informazioni sul modo in cui le persone ne parlano. La sua compilazione richiede indicativamente 20 minuti.

Di seguito troverà un elenco di parole che riguardano il dolore. Per ciascuna di esse esprima una valutazione seguendo le istruzioni che leggerà di volta in volta. Se non ha mai sentito una o più di queste parole, oppure se le ha già sentite ma non ne conosce il significato, barri la casella “NON CONOSCO”. Non abbia fretta nel compilarlo. ma cerchi di essere accurato/a e spontaneo/a.

Grazie.

*Good morning.*

*This questionnaire is part of a study on pain and aims to collect information on how people talk about it. Its completion requires approximately 20 minutes.*

*Below you will find a list of words associated to pain. Rate them following the instructions. If you have never heard one or more of these words, or if you have heard them but do not know exactly their meaning, check the “I DON’T KNOW” box. Do not hurry to complete it, but try to be accurate and spontaneous.*

*Thank you.*

## FAMILIARITÀ/*FAMILIARITY*

## Tra le parole che utilizziamo. ascoltiamo e leggiamo. ne esistono alcune che incontriamo molto spesso (estremamente familiari) e altre che non incontriamo quasi mai (per niente familiari). Per esempio, per la maggior parte delle persone la parola BICCHIERE è molto familiare, mentre la parola BALESTRA è poco familiare. Valuti per ciascuna delle seguenti parole quanto le è familiare in una scala che va da 1 (per niente familiare) a 7 (estremamente familiare).

## *Among the words we use, listen, and read, there are words that we encounter very often (extremely familiar) and other words that we almost never encounter (not at all familiar). For example, for most of the people the word GLASS is very familiar, while the word BALESTRA is not very familiar. For each of the following words, rate how familiar you are with them on a scale ranging from 1 (not at all familiar) to 7 (extremely familiar).*

## VALENZA EMOZIONALE*/EMOTIONAL VALENCE*

## La valenza emozionale descrive quanto il significato di una parola è negativo o positivo. Per esempio, per la maggior parte delle persone la parola ABBRACCIO è positiva e la parola GUERRA è negativa, mentre altre parole, come SEDIA, non sono né positive né negative. Valuti quanto negativo o positivo è per Lei il significato delle seguenti parole in una scala che va da -3 (estremamente negativo) a +3 (estremamente positivo).

## *The emotional valence describes how much a word’s meaning is negative or positive.*

## *For example, for most people the word HUG is positive and the word WAR is negative, while other words, like CHAIR, are neither positive nor negative. Rate how negative or positive the meaning of the following words is to you on a scale ranging from -3 (extremely negative) to +3 (extremely positive).*

## ATTIVAZIONE/*AROUSAL*

L'attivazione descrive quanto una parola si riferisce a qualcosa di molto attivante ed elettrizzante o, al contrario, a qualcosa che non attiva, che smorza o calma, indipendentemente che sia positiva o negativa. Per esempio, per la maggior parte delle persone le parole RELITTO e PANTOFOLA evocano un senso di inattivazione e di calma e le parole URAGANO e GAVETTONE evocano un senso di attivazione ed energia, mentre altre parole, come FILO, non evocano né un senso di attivazione, né un senso d’inattività. Valuti quanto le seguenti parole Le trasmettono attivazione ed energia oppure assenza di attivazione e assenza di energia in una scala che va da 1 (per niente attivante) a 7 (estremamente attivante).

*Arousal describes how much a word refers to something very activating and exciting or, on the contrary, to something that is not active, that dampens or calms, regardless of whether it is positive or negative.
For example, for most people the words WRECK and SLIPPER evoke a sense of inactivation and calm and the words HURRICANE and WATER BALLOON evoke a sense of activation and energy, while other words, such as THREAD, do not evoke neither a sense of activation, nor a sense of inactivity.
Rate how much the following words convey to you activation and energy or absence of activation and absence of energy on a scale ranging from 1 (not arousing at all) to 7 (extremely arousing).*

## QUANTO ASSOCIA LE SEGUENTI PAROLE AL DOLORE?/*HOW MUCH DO YOU ASSOCIATE THE FOLLOWING WORDS TO PAIN?*

Valuti per ciascuna delle seguenti parole quanto per lei sono associate al dolore inteso in senso ampio (sia fisico che psicologico) su una scala che va da 1 (per niente associata) a 7 (estremamente associata).

*Rate how much you associate the following words to pain in its broader meaning (both physical and psychological) on a scale ranging from 1 (not at all associated) to 7 (extremely associated).*

## INTENSITÀ/*INTENSITY*

Alcune delle parole che seguono possono descrivere diversi aspetti associati all'esperienza del dolore (sia fisico che psicologico). Valuti per ognuna l'intensità del dolore che descrive su una scala da 1 (per niente intensa) a 10 (estremamente intensa).

*Some of the following words may describe different aspects associated with the experience of pain (both physical and psychological). Rate the intensity of the pain they describe on a scale ranging from 1 (not at all intense) to 10 (extremely intense).*

## SPIACEVOLEZZA/*UNPLEASANTNESS*

Alcune delle parole che seguono possono descrivere diversi aspetti associati all'esperienza del dolore (sia fisico che psicologico). Valuti per ognuna la spiacevolezza del dolore che descrive su una scala da 1 (per niente spiacevole) a 10 (estremamente spiacevole).

*Some of the following words may describe different aspects associated with the experience of pain (both physical and psychological). Rate the unpleasantness of the pain they describe on a scale ranging from 1 (not at all unpleasant) to 10 (extremely unpleasant).*
